# Integrating cognitive behavioral therapy into music therapy methods using a flexibility within fidelity framework

**DOI:** 10.3389/fpsyt.2025.1734508

**Published:** 2025-12-09

**Authors:** John H. Head, Namrata N. Vasquez

**Affiliations:** 1 Arts in Medicine Research Lab, Houston Methodist Academic Institute, Center for Performing Arts Medicine, Houston Methodist, Houston, TX, United States; 2 Department of Psychiatry and Behavioral Health, Houston Methodist, Houston, TX, United States; 3 Houston Methodist Research Institute, Houston, TX, United States; 4 Weill Cornell Medicine, New York, NY, United States

**Keywords:** music therapy, psychotherapy, cognitive behavioral therapy, flexibility within fidelity, mental health

## Abstract

Cognitive behavioral therapy (CBT) is one of the most researched evidence-based practices in mental health treatment. While studies have examined the effectiveness of music therapy in comparison or in addition to cognitive behavioral therapy, none have examined how to use principles of cognitive behavioral therapy within the four music therapy methods using flexibility within fidelity as a framework. A real case example is provided to illustrate the incorporation of CBT principles into music therapy methods resulting in resolution of PTSD and anxiety symptoms.

## 1 Introduction

Cognitive behavioral therapy (CBT) is one of the most researched evidence-based practices in mental health treatment (1). In music therapy there is a rich tradition of differing perspectives in mental health care (e.g., humanistic and psychodynamic) but little about music therapy and CBT (2).While studies have examined the effectiveness of music therapy in comparison or in addition to cognitive behavioral therapy, none have examined how to use principles of cognitive behavioral therapy within the four broad music therapy methods as defined by Bruscia (2) using the concept of flexibility within fidelity (3). Incorporating CBT principles into music therapy methods can facilitate both musical engagement and cognitive restructuring to create an evidence-based, individualized, and culturally informed approach to treatment.

## 2 Evidence-based practice and treatment

Evidence-based practice (EBP) brings together the best available research, clinical expertise, and patient characteristics, culture, and preferences to guide psychological care (APA, n.d.; 4). At its core, EBP supports treatment strategies and relationships that are both scientifically validated and contextually meaningful for the person. Collaboration is essential, and EBP encourages shared decision-making between clinician and client that is grounded in data, clinical insight, and available resources. The goal is improved care that stays rooted in effective principles while adapting to the individuality of each client.

### 2.1 Flexibility within fidelity

When integrating evidence-based treatments into music therapy practice—or any individualized mental health work—providers face a familiar challenge: how do we honor the integrity of the treatment while also tailoring it to the person? Kendall’s Flexibility within Fidelity (FWF) framework asks therapists to first identify and preserve the treatment’s core components—responsible for therapeutic change—while flexibly adapting select elements (the adaptable periphery) to fit client needs, preferences, and culture (3). Adaptations might include adapting homework that is common in CBT to in-session music therapy methods, shifting session structure, or integrating culturally relevant metaphors and experiences —but only if the core components stay in place. This approach supports the rigor of evidence-based care and the flexibility needed for meaningful application. The FWF model doesn’t diminish the need for training. Therapist adherence and competence remain crucial to clinical outcomes, even within adaptive frameworks (Webb et al., 2010).

## 3 Cognitive behavioral therapy

CBT is among the most widely studied and practiced evidence-based approaches in mental health care, with demonstrated effectiveness across a range of diagnoses including anxiety, depression, post-traumatic stress disorder, and eating disorders (1). CBT posits that automatic thoughts arise in response to antecedent events and shape emotional, behavioral, and physiological reactions. When maladaptive, these thoughts can reinforce psychological distress and dysfunctional coping. The A-B-C model—activating event, belief, and consequence—maps the cognitive and emotional sequences that drive these outcomes (5–7).

Over time, clients often begin to recognize underlying core beliefs—deeply rooted assumptions shaped by earlier experiences—that influence their interpretations and behaviors. These beliefs, along with their associated conditional rules, can become rigid and self-defeating. CBT uses structured interventions such as cognitive restructuring, behavioral activation, and exposure to help clients develop more adaptive thinking and behavior patterns (5).

While CBT is structured, it is not rigid. The FWF framework emphasizes preserving the theoretical integrity of core treatment components while allowing adaptation of form, order, or medium (e.g., musical engagement) to meet individual needs (3). Similarly, flexible treatment and exposure pacing in CBT for depression and youth anxiety has been associated with improved outcomes (8, 9; Strunk et al., 2022).

Implementation research reinforces this view: when therapists make principled adaptations based on clinical reasoning rather than convenience, outcomes often improve (10, 11). In trauma-focused work, core CBT elements like exposure and cognitive processing can be preserved while creatively tailoring how clients access and express traumatic material (12). The FWF model strives for a middle ground between rigid fidelity and loose improvisation.

This conceptual flexibility opens the door for thoughtful integration of CBT within other disciplines—particularly music therapy.

## 4 CBT in relation to music therapy

Music therapy has been studied in various contexts alongside CBT—including as a standalone intervention, an adjunct to CBT, and as a CBT-informed approach within music therapy sessions—across diverse populations and international settings (13–17). Findings suggest that, in some cases, combining music therapy with CBT enhances symptom improvement beyond CBT alone (16), while in others, CBT-informed group music therapy yields outcomes comparable to standard CBT groups (15, 18, 19).

These studies raise a conceptual issue: is it appropriate to position music therapy and CBT as distinct, directly comparable treatments? CBT is a structured treatment model grounded in specific psychological mechanisms and learning theory, whereas music therapy is a clinical profession that encompasses a range of orientations and techniques. Comparing the two is like comparing a profession (e.g., counseling) to a single therapeutic modality (e.g., CBT). Rather than asking which is more effective and conceptualizing CBT and music therapy as mutually exclusive treatment modalities, music therapists might explore how to meaningfully integrate well-researched evidence-based modalities such as CBT principles into their work while preserving the identity and strengths of their practice, namely musical engagement.

Hakvoort (2014) developed Cognitive Behavioral Music Therapy within forensic psychiatry, demonstrating how CBT principles such as coping skill training and anger management could be embedded in structured music therapy programs. Her work highlights the feasibility of CBT-informed music therapy in a specific context, which this paper expands by applying the FWF model to integrate CBT more broadly across music therapy methods.

The FWF model offers a framework for adapting the mode of delivery to fit the client’s needs through the medium of music. It shifts the question from “Can music therapy compete with CBT?” to “How can music therapists embed cognitive-behavioral theory into the music therapy methods they already use?” When fundamental CBT principles are adapted to music therapy practice, music therapy interventions can encourage cognitive restructuring through an embodied, real-time, culturally informed, and emotionally resonant form of feedback that supports meaningful psychological change.

## 5 CBT techniques within music therapy methods

Music therapy methods, categorized by Bruscia (2) as receptive, compositional, improvisational, and re-creative, can be applied to any therapeutic approach (e.g., psychodynamic, behavioral, humanistic). Using FWF, music therapists may flexibly implement CBT techniques into any of these existing methods. While CBT usually requires the client to complete homework to foster autonomy, in music therapy it is also possible to collaboratively work through automatic thoughts, devise new alternative thoughts, and observe the results through live musical experiences, allowing clients to immediately experience cognitive restructuring through musical feedback. The following explanation of the music therapy methods is supported by a real case example that demonstrates one music therapist’s applications of CBT principles within all four music therapy methods.

### 5.1 Case example

B, a White male nurse in his early thirties, entered treatment for moderate and worsening anxiety marked by panic attacks and chronic stress related to workplace trauma, ongoing exposure to death, and a close family member’s recent medical emergency. These experiences, coupled with a recent family loss, intensified his mortality salience and distress around his role as a caregiver. Both professionally and personally, B’s self-perceived caregiver identity reinforced perfectionism, hypervigilance, and self-critical thought patterns, all of which contributed to psychological rigidity and impaired coping.

B’s treatment team included a clinical psychologist, a psychiatric nurse practitioner, and a board-certified music therapist. His goals were improving emotional regulation, reducing panic symptoms, and gaining insight into his anxiety. Across providers, cognitive-behavioral techniques and mindfulness-based strategies were used to support these aims. The music therapist integrated CBT principles into music-based interventions, adapting them across receptive, compositional, improvisational, and re-creative methods to incorporate B’s musical engagement and therapeutic goals.

### 5.2 Receptive

Receptive music therapy describes an umbrella of interventions in which music listening is the form of musical engagement (2). In receptive music therapy informed by CBT, listening to music functions as the activating event in the A-B-C model, prompting automatic thoughts, emotional responses, and physiological cues. Personally meaningful or preferred music can evoke vivid memories, embedded beliefs, personality traits, attachment styles, and ingrained emotional patterns (20–23). These are entry points for therapeutic processing, where clients can identify underlying beliefs and their emotional or behavioral consequences.

Receptive methods were integral in the early phase of B’s treatment. During his assessment, he reported a strong connection to Sturgill Simpson’s music, whose lyrics explore existential themes and inner conflict. B selected a Simpson track for listening and reflective discussion. B identified core values expressed in the lyrics—personal responsibility, emotional honesty, and autonomy—and began to explore how these themes mirrored his own struggles with self-worth and emotional restraint.

This process laid the foundation for cognitive restructuring. Inspired by the session, B voiced a desire to rewrite the lyrics. As he shifted into compositional work, he began reauthoring his internal narrative, translating insight into action by replacing rigid, self-critical beliefs with more compassionate and flexible alternatives.

### 5.3 Compositional

Music composition invites clients to make intentional choices—about lyrics, instrumentation, melody, rhythm, and harmony—that reflect both cognitive processes and emotional states (2). Lyrical content typically conveys explicit thoughts and emotions, while musical elements provide implicit emotional and cultural context. Thus, cognitive and affective material can be evoked, expressed, and integrated within one intervention.

CBT concepts naturally align with the songwriting process. For instance, the A-B-C model can shape a song’s structure: verses depict activating events (A), choruses express automatic beliefs (B) and their emotional consequences (C), and bridges offer restructured thoughts formed through Socratic questioning or guided reappraisal (24). This format transforms songwriting into a creative form of cognitive restructuring and exposure, allowing clients to externalize internal conflicts and reinforce new perspectives through performance, revision, and repetition.

In B’s case, songwriting became a central strategy for processing his panic symptoms. He composed a piece based on Sturgill Simpson’s *All Around You* that began as an unfiltered expression of distress. B and the therapist discussed his lyrics using Socratic questioning and identified where automatic thoughts emerged. He revised these lines to reflect more balanced and empowering beliefs, grounded in insights from receptive and verbal processing. This act of re-writing allowed B to encode new cognitive patterns in a form he could rehearse emotionally and musically.

Therapists may help clients integrate CBT into songwriting in varied ways. Some map the A-B-C model directly onto a song’s structure; others embed lines of Socratic questioning or value clarification into their lyrics. These creative strategies preserve CBT’s therapeutic mechanisms while translating them into emotionally resonant, culturally relevant, and personally meaningful interventions. Composition is a deliberate practice which makes time for reflection and revision. Improvisation, on the other hand, requires intuitive responses to musical engagement.

### 5.4 Improvisational

In improvisational music therapy, music is created spontaneously with or without established rules and themes (2). Improvisation can provide immediate access to a client’s internal processes. Within a CBT-informed approach, spontaneous music-making becomes the activating event (A), provoking automatic thoughts (B) and emotional consequences (C). B once described frustration from “making mistakes” during improvisation, stating that his playing on the piano did not sound like the music he enjoyed. Such comments revealed perfectionistic beliefs beyond his music-making. B and the therapist replayed recordings from their piano improvisations to uncover thoughts like “I should have known this” and “This should be better.” The therapist responded with Socratic questioning: “Why should this be good?” “Tell me the last time you practiced improvisation at all, let alone on the piano”. Over time, B began recognizing and replacing these rigid beliefs with more adaptive ones (e.g., “it doesn’t have to sound perfect”). The therapist provided guidance for further exploration about his perfectionism outside the session, and ways to apply his reshaped musical experience to his lived experience.

B became more willing to take risks, explore unfamiliar instruments and sounds, and tolerate uncertainty, with music as a rehearsal space for cognitive flexibility and emotional resilience.

### 5.5 Re-creative

Re-creative music therapy uses pre-existing music (2). Re-creation often evokes more than just technical challenges—it can stir deeply held beliefs shaped by earlier experiences. Many clients recall moments when someone important emphasized perfection over exploration. These moments, though brief, can solidify into enduring thoughts (e.g., “I’m not good at anything,” always mess up.). As clients encounter small mistakes (e.g., a missed note), their internal narrative may quickly spiral: “I played a wrong note” becomes “I did it wrong because I’m wrong” This distortion reflects a common shift from behavioral guilt to global shame.

CBT’s A-B-C model offers a framework to deconstruct these reactions. The activating event (A) might be a musical slip; the belief (B), a self-critical thought; and the consequence (C), overall shame and avoidance. By decelerating this process in session, therapists can help clients notice automatic thoughts and try alternatives. For example, a client might experiment with reframing— “It’s okay to make mistakes while learning”—or engage in behavioral tasks like replaying the passage slowly with observations rather than judgment.

For B, learning guitar allowed him to challenge perfectionistic thinking in real time. Initially hesitant, he began practicing regularly and gradually noticed a shift in how he spoke to himself. Instead of quitting after mistakes, he developed more curiosity and persistence. His therapist noted improved emotional regulation and performance confidence, which B attributed in part to growing self-compassion. He noted that when he stopped shaming himself for mistakes, he improved and felt more relaxed.

B’s experiences bridged music and daily life as real-time sensory feedback reinforced new thought patterns in an immediate, embodied way. With a more relaxed mental stance, he often produced clearer tone and steadier rhythm—proving that shifts in thinking can be not just heard but felt.

## 6 Data collection

Client data were collected using the Generalized Anxiety Disorder 7 (GAD-7) and the PTSD Checklist for DSM-5 (PCL-5), two validated and widely used screening tools for generalized anxiety disorder and post-traumatic stress disorder (PTSD) (25, 26). Data was collected monthly with one exception due to provider absence using a collaborative approach; the team partnered with the client to determine which PROs best captured his experiences and symptoms.

## 7 Results

B scored a 39 on the PCL-5 at baseline and endorsed at least one item for each PTSD criterion. His GAD-7 score was 11, suggesting moderate anxiety. Throughout treatment, all of B’s symptoms initially showed a decline that was then followed by a spike. The spike was attributed to his disclosure of relationship issues that exacerbated feelings of shame related to perfectionism. His PCL-5 scores, when broken down into criteria, showed this notable increase in negative alterations in cognition and mood. As treatment progressed, the client’s scores resumed a steady decline, scoring 8 (no PTSD symptoms present) on the PCL-5 and 6 (below the mild cutoff) on the GAD-7 at the end of treatment.

Over 14 sessions, B’s demonstrated increasing openness and self-acceptance. After B tapered treatment with subclinical mild anxiety symptoms, he became open to pharmacological treatment, improved his psychological flexibility, and participated in more confident decision-making. B reported greater effectiveness at work, more meaningful engagement with hobbies and nature, and greater relationship satisfaction, including more effective communication with his partner. B viewed the future with a hopeful outlook and confidence that he could cope with stressors. B’s improved PROs suggest that treatment was effective in managing his posttraumatic stress and anxiety symptoms.

## 8 Discussion

Evidence-based practices in clinical care are central to improving client outcomes and maintaining high standards in mental health services. The “flexibility within fidelity” (FwF) framework offers one approach to doing so while still responding to individual client needs, background, and clinical contexts.

This case demonstrated one way that CBT principles—especially those derived from the A-B-C model—were applied across the four music therapy methods: receptive, compositional, improvisational, and re-creative to address a client’s specific psychological treatment goals. Through each CBT-informed music therapy method, the client engaged with music in ways that evoked automatic thoughts and emotional consequences to support cognitive restructuring.

This case does not add new acronym-ed therapy to the already bursting pantheon of mental health therapies but illustrates how therapists might adapt CBT content to music therapy methods while maintaining its foundational principles. It complements Hakvoort’s (2014) CBMT model but uses FWF as a transdiagnostic framework rather than a standalone treatment model. The adapted approach showcases how music therapy methods offer real-time, sensory feedback that reinforces adaptive thought patterns and coping strategies. Future work might further explore how fidelity to CBT’s core components can be preserved within the unique relational and creative frameworks of music therapy practice.

## 9 Limitations

Integrating evidence-based practices into music therapy presents several challenges. Chief among them is the need for music therapists to receive appropriate training in the theoretical underpinnings and delivery of treatments like CBT. Without adequate preparation, therapists risk deviating from the core principles that make such interventions effective. In the case presented, integration of CBT techniques was made possible through collaboration with professionals with these specialties; collaboration helps teams develop more cohesive and effective treatment plans but may be limited in settings where interdisciplinary communication does not have the same level of structural and administrative support.

Additionally, while the FWF framework supports individualization, it also demands clear understanding of what constitutes an intervention’s core components and what can be adapted. More research is needed to examine how these principles function in practice when applied to music therapy methods and to identify effective, necessary adaptations.

This case is a detailed example of how CBT principles can be meaningfully integrated into music therapy and not a substitute for controlled trials or broad-based implementation studies. Findings were limited by single-case design and reliance on clinician-reported process and client-reported outcomes. Future studies should investigate the replicability of these strategies across diverse clients, settings, and therapists.

## Data Availability

The raw data supporting the conclusions of this article will be made available by the authors, without undue reservation.
